# Association between deep gray matter iron deposition and clinical symptoms in Parkinson’s disease: a quantitative susceptibility mapping study

**DOI:** 10.3389/fneur.2024.1442903

**Published:** 2025-01-06

**Authors:** Hui Zhao, Qiu-Hong Ji, Zhong-Zheng Jia, Li-Hua Shen

**Affiliations:** ^1^Department of Neurology, Affiliated Hospital of Nantong University, Medical School of Nantong University, Nantong, China; ^2^Department of Neurology, Affiliated Rudong Hospital of Xinglin College, Nantong University, Nantong, China; ^3^Department of Medical Imaging, Affiliated Hospital of Nantong University, Medical School of Nantong University, Nantong, China

**Keywords:** Parkinson’s disease, iron deposition, quantitative susceptibility mapping, motor, non-motor

## Abstract

**Purpose:**

This study aimed to assess the association between motor and non-motor symptoms of Parkinson’s disease (PD) and iron accumulation within the deep gray matter of the brain by Quantitative Susceptibility Mapping (QSM).

**Methods:**

Fifty-six PD patients and twenty-nine healthy controls were recruited in this study. According to the Hoehn and Yahr (H-Y) stage score, PD patients were divided into early stage (H-Y ≤ 2) and advanced stage (H-Y > 2) groups. Specifically, the Regions of Interest (ROIs) encompassed the substantia nigra (SN), red nucleus (RN), caudate nucleus (CN), globus pallidus (GP) and putamen (PT). Meanwhile, various rating scales were used to assess the clinical symptoms of PD.

**Results:**

Compared to healthy controls (HCs), PD patients showed a significant increase in magnetic susceptibility values (MSVs) within the SN and GP. Further comparisons indicated that the MSVs of the SN, PT, GP and CN are all higher in advanced stages than in early stages. Significant positive correlations were observed between the MSVs of the SN and scores on the UPDRS-III, HAMA, and HAMD (*r* = 0.310, *p* = 0.020; *r* = 0.273, *p* = 0.042; *r* = 0.342, *p* = 0.010, respectively). Likewise, the MSVs of the GP demonstrated notable correlations with HAMA and HAMD scores (*r* = 0.275, *p* = 0.040; *r* = 0.415, *p* = 0.001). Additionally, a significant correlation was found between the MSVs of the PT and HAMD scores (*r* = 0.360, *p* = 0.006). Furthermore, we identified a significant negative correlation between MMSE scores and the MSVs of both the PT and GP (*r* = −0.268, *p* = 0.046; *r* = −0.305, *p* = 0.022).

**Conclusion:**

Our study revealed that QSM possesses the capability to serve as a biomarker for PD. Significant correlations were found between clinical features and the iron deposition in the nigrostriatal system.

## 1 Introduction

Parkinson’s disease (PD) is a common neurodegenerative disease that affects approximately 2–3% of the world’s population aged 65 years and over (1), and is characterized by the depletion of dopaminergic neurons in the substantia nigra (SN), leading to a subsequent dopamine deficiency in the striatum (2). PD is primarily known for its motor impairments caused by the gradual depletion of dopaminergic neurons. Nonetheless, individuals with PD also endure a wide range of disabling non-motor symptoms, such as anosmia, constipation, autonomic dysfunction, psychiatric manifestations and cognitive impairment (3), these symptoms are not directly linked to the degree of motor impairment and may even manifest prior to the appearance of motor symptoms.

Over the past few years, significant progress has been made by researchers in understanding the pathology of the disease. Although the precise mechanisms underlying PD pathology are complex and involve multiple factors, several molecular pathways have been identified as playing a role, such as the accumulation of misfolded *α*-synuclein, mitochondrial dysfunction, oxidative stress, inflammation, and elevated iron levels (4–7).

Research has demonstrated that iron plays a pivotal role in the cell death process known as ferroptosis (8), and iron deposition has long been suspected as a contributing factor in the damage of dopaminergic neurons (9). The accumulation of iron in the brains of healthy individuals usually increases with age (10), but this increase is notably less pronounced compared to that seen in patients with PD. As a powerful pro-oxidant, excess iron causes an increase in reactive oxygen species, disrupts mitochondrial function, and ultimately leads to cell death through iron-related mechanisms (11, 12). Furthermore, iron overload can facilitate the aggregation of *α*-synuclein (6). Quantitative susceptibility mapping (QSM) is a Magnetic Resonance Imaging (MRI) technique that can be employed to quantify iron content (13). Anatomical landmarks, particularly those found in deep gray matter regions, can be easily recognized using QSM. QSM exhibits increased precision in measuring iron concentrations in tissues, coupled with improved dependability and reproducibility (14). Additionally, the magnetic susceptibility values (MSVs) obtained through QSM provide useful quantifiable metrics for evaluations and comparisons among different groups.

In this study, we used QSM to determine iron content in the deep gray matter of PD brain, with the aim of evaluating iron deposition in different stages of PD and its association with motor and non-motor symptoms of PD.

## 2 Materials and methods

### 2.1 Participants and agreements

From November 2021 to November 2023, a total of 56 patients were consecutively recruited from the Department of Neurology, Affiliated Hospital of Nantong University. The diagnosis of PD was made according to the MDS clinical diagnostic criteria for idiopathic PD (15) and was evaluated by two neurologists specializing in movement disorders. Additionally, 29 healthy controls (HCs), matched for age and gender, were included in the study, with no known history of clinically overt neurological or psychiatric disease. Patients were excluded from the study if they presented with the following conditions: (1) secondary parkinsonism, parkinson-plus syndromes and hereditary parkinson syndromes; (2) a history of neurological diseases, such as severe head trauma or stroke; (3) poor image quality; (4) general contraindications for MRI scanning, including claustrophobia, pacemaker, or implanted metal parts. (5) PD patients had comorbidities that could influence iron accumulation or clinical symptom expression. The Ethics Committees of the Affiliated Hospital of Nantong University granted approval for this study, and prior to participation, written informed consent was obtained from all subjects.

### 2.2 Clinical evaluations

All patients with PD were assessed within 1 week before QSM by a series of rating scales for evaluating the motor and non-motor symptoms. Severity of PD was assessed by Hoehn and Yahr (H-Y) stage scores, and patients with a stage ≤2 were grouped into the early stage PD group, while those with a stage >2 were grouped into the advanced stage PD group. The third part of the MDS-Unified PD Rating Scale (UPDRS-III) (16), served as the metric to determine the extent of motor symptoms. Additionally, the Mini-Mental Status Examination (MMSE) was used to assess cognitive impairment, while the Hamilton Anxiety Scale (HAMA) and the Hamilton Depression Scale (HAMD) were utilized to measure anxiety and depression, respectively.

### 2.3 MRI acquisition

All participants underwent MRI scans using a 3 T MR scanner (Signal 750w; GE Healthcare, USA) with a multi-echo gradient recalled echo (GRE) sequence. All PD patients discontinued dopamine agonists for one week prior to the scan. To prevent head movement and minimize scanner noise, foam pads and earplugs were used. In addition to the GRE sequence for QSM, routine brain MRI scans included T1-weighted imaging (T1WI), T2-weighted imaging (T2WI), and T2-weighted fluid attenuated inversion recovery (T2-FLAIR), which would be helpful for anatomical guidance and lesion exclusion. The scan parameters for QSM were set as follows: echo time (TE) of first echo = 3.3 ms; echo spacing = 2.3 ms; total number of echoes = 16; repeat time (TR) = 32.5 ms; flip angle (FA) = 20°; field of view (FOV) = 256 × 256 mm^2^; matrix = 256 × 256; layer thickness = 1 mm; acceptance bandwidth = 62.50 Hz/Px; imaging time = 3 min 42 s.

### 2.4 MRI post processing

**Figure 1:**
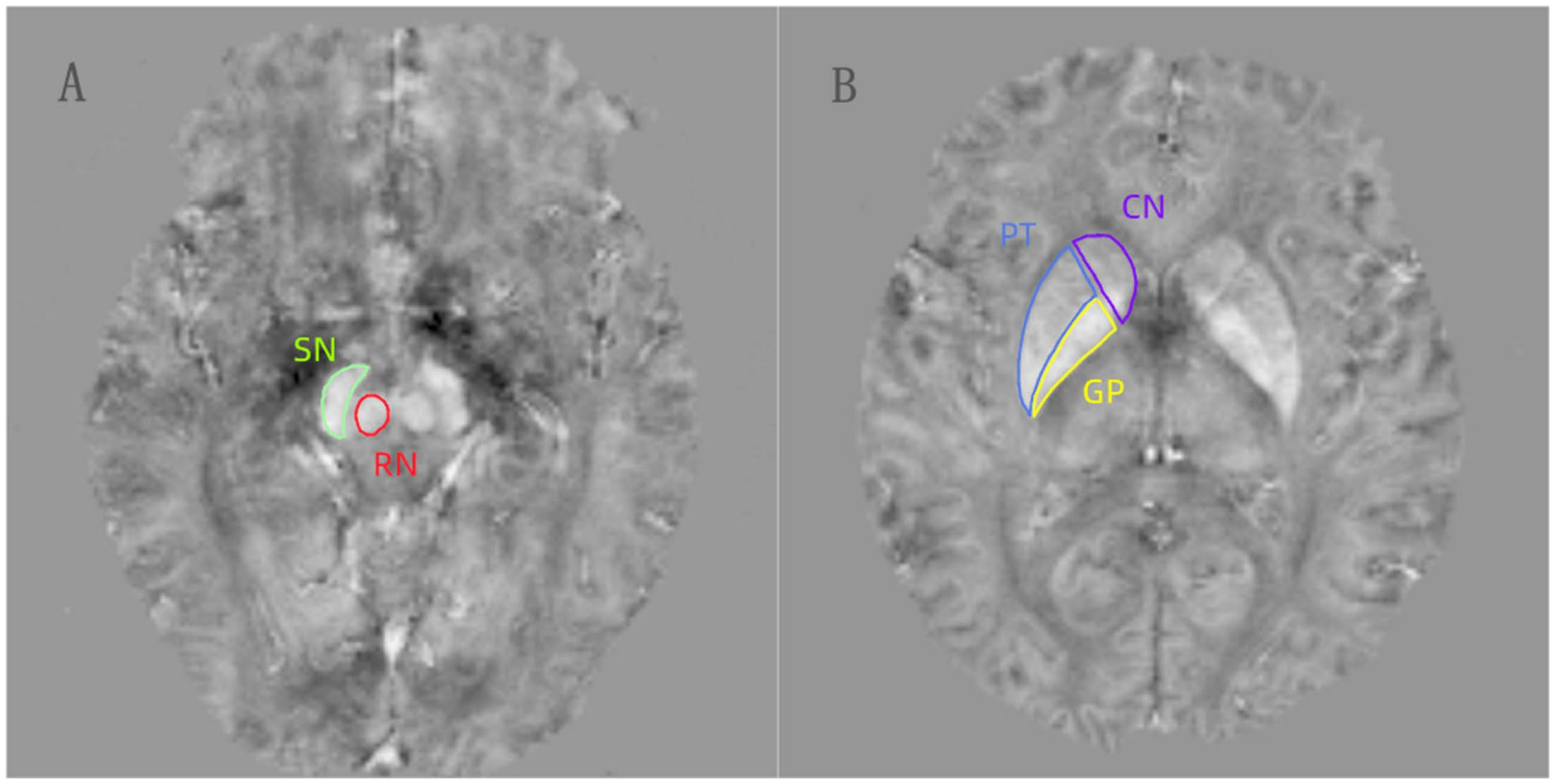
**(A,B)** The regions of interests (ROIs) were manually drawn for the substantia nigra (SN), red nucleus (RN), globus pallidus (GP), putamen (PT), and caudate nucleus (CN) on the QSM images.

The phase images with multiple echoes were unwrapped using a Laplacian-based unwrapping technique (17). To eliminate the background field from the unwrapped phase image, the V-SHARP (variable-kernels sophisticated harmonic artifact reduction for phase data) method was applied in conjunction with a binary brain mask (18). Additionally, we utilized the STAR-QSM technique to further reduce streaking artifacts in the image (19). The QSM scans encompassed the bilaterally symmetric regions of the substantia nigra (SN), red nucleus (RN), globus pallidus (GP), putamen (PT) and caudate nucleus (CN) (Figure 1). Cerebrospinal fluid was chosen as the reference region for QSM of the brain. Two experienced neuroradiologists, blinded to the subjects’ identity and clinical details, manually traced the ROIs using AW Volume Share 5.0 software. First, we opened the QSM images in the software and adjusted the contrast of the acquired images. Then, we selected the most prominent layer, along with two adjacent layers of each nucleus, and performed manual segmentation. Finally, the software performed additional calculations to determine the average one-sided MSV of the three layers. Subsequently, the mean values from both sides were utilized as regional MSVs for further analysis. The unit for these MSVs is ppm (parts per million).

### 2.5 Statistical analysis

All datas analyzed in this study were processed using IBM SPSS 25.0 software. Normality tests were conducted on the continuous variables. Variables exhibiting a normal distribution were presented using mean ± standard deviation. Variables that deviated from a normal distribution, on the other hand, were represented by the median and interquartile range. Categorical variables were expressed as frequencies and percentages. In this study, age, UPDRS-III scores, HAMA scores, and regional MSVs all exhibited a normal distribution, while the disease duration, HAMD scores, and MMSE scores all followed a skewed distribution.

For continuous variables, the two datasets were compared using either the independent samples *t*-test (when the data followed a normal distribution) or the Mann–Whitney U-test (when the distribution was skewed). Pearson’s chi-square tests were used to compare categorical variables. Spearman or pearson correlation analysis was used to evaluate the correlations between MSVs and clinical scores. *p* < 0.05 was considered to be statistically significant.

## 3 Results

### 3.1 Demographic data

Demographic and clinical characteristics were summarized in Table 1. Compared to the HCs, the PD group exhibited statistically significant differences in the MSVs of the SN and GP, and the intercomparisons of MSVs between the two groups were performed controlling for age and gender. Whereas, no significant differences were observed in the MSVs of the RN, PT, and CN.

**Table 1:** Baseline demographic and clinical characteristics of participants.

	PD (*n* = 56)	HCs (*n* = 29)	*p*
Gender (male, %)	32 (57.1%)	15 (51.7%)	0.634
Age (years)	63.04 ± 8.66	62.07 ± 10.36	0.650
SN	0.071 ± 0.026	0.055 ± 0.019	0.001
RN	0.094 ± 0.027	0.089 ± 0.022	0.390
PT	0.094 ± 0.024	0.086 ± 0.016	0.097
GP	0.140 ± 0.037	0.116 ± 0.020	<0.001
CN	0.063 ± 0.019	0.057 ± 0.020	0.189

PD, Parkinson’s disease; HCs, healthy controls; SN, substantia nigra; RN, red nucleus; GP, globus pallidus; PT, putamen; CN, caudate nucleus. The unit of MSVs is ppm.

### 3.2 Comparison of MSVs in different brain ROIs between early PD and advanced PD

Table 2 summarizes the comparison between PD patients with early and advanced disease stages, indicating that patients in the advanced stage had higher ages, longer disease durations, and higher scores on the UPDRS-III, HAMA, and HAMD compared with patients in the early stage. Furthermore, significant differences could be seen when referring to iron deposition in SN, PT, GP, and CN, which is compliant with the progressiveness of PD. However, no marked differences in MSVs of RN were found between the two groups.

**Table 2:** Comparisons of clinical characteristics and regional MSVs between Early PD and Advanced PD.

	Early PD (*n* = 32)	Advanced PD (*n* = 24)	*p*
Gender (male, %)	19 (59.4)	13 (54.2)	0.697
Age (years)	61.28 ± 8.46	65.38 ± 8.54	0.080
Duration (years)	2.00 (0.85–3.00)	7.50 (5.00–9.75)	<0.001
UPDRS-III	12.00 (9.00–17.75)	30.50 (21.50–39.25)	<0.001
MMSE	26.00 (25.00–28.00)	26.00 (24.00–27.00)	0.242
HAMA	10.94 ± 4.18	13.71 ± 4.84	0.026
HAMD	4.00 (2.00–7.00)	9.50 (7.25–12.75)	<0.001
SN	0.065 ± 0.023	0.079 ± 0.028	0.035
RN	0.094 ± 0.028	0.094 ± 0.025	0.996
PT	0.087 ± 0.186	0.103 ± 0.028	0.023
GP	0.131 ± 0.032	0.153 ± 0.040	0.024
CN	0.058 ± 0.018	0.070 ± 0.018	0.024

PD, Parkinson’s disease; SN, substantia nigra; RN, red nucleus; GP, globus pallidus; PT, putamen; CN, caudate nucleus; UPDRS-III, the third part of the MDS-Unified PD Rating Scale; HAMA, Hamilton Anxiety Scale; HAMD, Hamilton Depression Scale; MMSE, Mini-Mental Status Examination. The unit of MSVs is ppm.

### 3.3 Correlation analysis between clinical characteristics and regional MSVs in patients with PD

**Figure 2:**
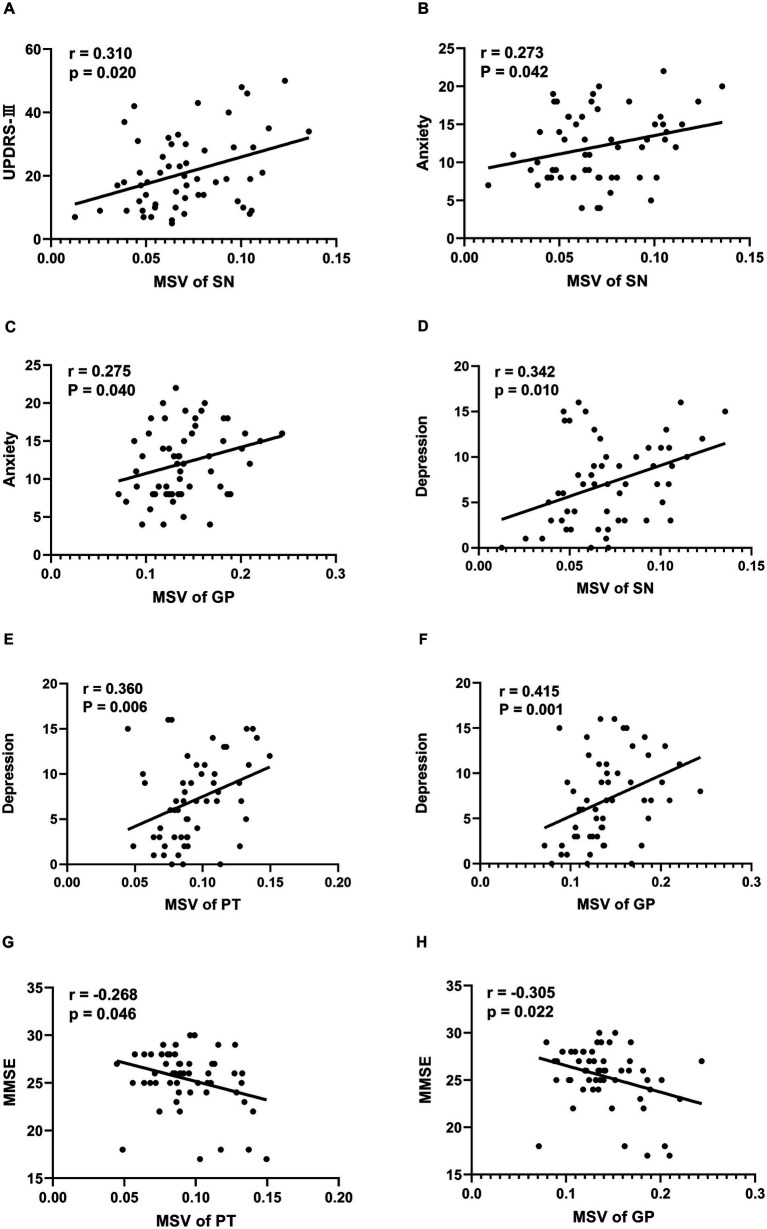
Correlation between clinical Characteristics and regional MSVs in PD patients. **(A)** Correlation between UPDRS-III scores and MSVs of SN. **(B)** Correlation between anxiety scores and MSVs of SN. **(C)** Correlation between anxiety scores and MSVs of GP. **(D)** Correlation between depression scores and MSVs of SN. **(E)** Correlation between depression scores and MSVs of PT. **(F)** Correlation between depression scores and MSVs of GP. **(G)** Correlation between MMSE scores and MSVs of PT. **(H)** Correlation between MMSE scores and MSVs of GP. The unit of MSVs is ppm.

In the PD patients recruited for the current study, Table 2 and Figure 2 demonstrate a significant correlation between regional MSVs and the scores of the UPDRS-III, HAMA, HAMD, and MMSE. The results turned out that the MSVs of SN were significantly and positively correlated with scores of UPDRS-III, HAMA and HAMD (r = 0.310, *p* = 0.020; r = 0.273, *p* = 0.042; r = 0.342, *p* = 0.010, respectively). Similarly, MSVs of GP also had a notable correlation with scores of HAMA and HAMD (r = 0.275, *p* = 0.040; r = 0.415, *p* = 0.001). And the MSVs of PT was significantly correlated with the HAMD scores (r = 0.360, *p* = 0.006). We also observed a significant negative correlation between the MMSE scores and the MSVs of PT and GP (r = −0.268, *p* = 0.046; r = −0.305, *p* = 0.022).

## 4 Discussion

Until now, the precise contributing factors in the development of PD remain elusive. It has been shown that in patients with PD, excess iron deposition has been observed in the deep gray matter structures of the brain, including the basal ganglia (comprising the GP, PT, and CN), the midbrain (including the RN and SN), and the dentate nucleus. Using QSM analyses to map the MSV in the nigrostriatal system, we found increased iron accumulation in SN and GP in PD patients compared with the HCs. As the disease progressed into the advanced stages, iron deposition extended to be more severe in the SN, GP, PT, and CN regions than in the early stages.

Several studies have documented the presence of unusual iron accumulations in certain deep brain nuclei of individuals with PD (20, 21). However, there is some inconsistency in these findings, which might stem from the limited sensitivity of the detection techniques and the relatively small number of participants. Studies have indicated that the SN is a crucial structure that has a significant impact on the pathophysiology of individuals with PD (22). A study utilizing QSM demonstrated that the concentration of iron in the SN of PD patients is significantly increased, exceeding the normal levels found in HCs. Furthermore, PD patients who exhibit more severe iron accumulation in the SN tend to score higher on the UPDRS motor section (20), which is consistent with our study.

The process of iron deposition in PD is complex, encompassing various mechanisms that affect iron distribution, transportation, storage, and circulation, ultimately resulting in iron accumulation within the SN and other parts of the brain (6). Previous studies have indeed demonstrated the existence of a diverse array of neural circuits, projections, and interconnected neural networks between the SN and the striatum. The dysfunction of dopaminergic striatal pathways is intricately linked to the elevated iron content in the SN (23). The cortical–limbic-striatal circuitry is also a functional brain network involved in neuromodulation. These intricate connections play a crucial role in the regulation and modulation of various motor and non-motor functions in the brain (24).

The potential associations between non-motor symptoms in PD and the underlying nigrostriatal pathology are not well understood. Increasing evidence suggests that a nigrostriatal dopamine deficiency in PD is linked not only to motor symptoms but also to non-motor symptoms (25, 26). Our study revealed that there is a notable correlation between iron deposition within the deep gray matter and non-motor symptoms (including anxiety, depression, and cognitive impairment), which was less frequently discussed in previous studies.

Anxiety is an emotional state that can arise from the anticipation of a potential or envisioned future danger or threat (27). Dopamine is a crucial neurotransmitter found in the central nervous system and plays a significant role in regulating human emotions. The degeneration of dopaminergic neurons located in the SN is confirmed to be the main pathological feature of PD, and alterations in dopaminergic activity within the limbic cortico-striato-thalamocortical circuits might also explain the high prevalence of anxiety among PD patients (28). Elevated iron levels within the neural circuits linked to fear in the brain are associated with anxiety in PD (29). Avila et al. (30) indicated that dopamine interacting with D2 or D4 receptors in the GP might play a role in the manifestation of anxiety. Our study revealed that PD patients with greater iron deposition in the SN and GP exhibited more severe anxiety symptoms. This iron accumulation contributes to cellular and tissue damage, potentially explaining the development of anxiety in PD.

Correlation analysis in our study provided some objective evidence that iron deposition within the SN, GP, and PT was actually correlated with the depression symptoms of PD patients. PD patients with depression exhibited a more significant loss of dopaminergic neurons in the SN compared to those without depression (31). This suggests that the SN is a crucial area for mood modulation in patients with PD (32). Moreover, in PD patients with depression, there is a noticeable widespread degeneration of dopaminergic terminals specifically within the striatum, and more prominently in the dorsal caudate nucleus (33). Hamilton et al. (34) observed that the raclopride binding potential increased in the ventral striatum bilaterally and in the right dorsal striatum of depressed participants. Furthermore, the connectivity between these regions and cortical targets, such as the cortico-striatal-pallido-thalamic circuit, was decreased.

Dopamine also plays a pivotal role in modulating hippocampal-dependent mnemonic processes, exerting distinct effects on multiple facets of memory and cognition (35). A previous study showed that striatal iron accumulations were correlated with dopaminergic deficits and neurophysiological signs in patients with PD (36). Our study found that there is a positive correlation between the iron deposition in the GP, PT and the severity of cognitive impairment in patients with PD. A recent study revealed that age-related alterations occur in the dopaminergic innervation of the striatum, resulting in decreased cognitive performance (37). Stögbauer et al. (38) found that the degeneration of dopaminergic neurons in the striatum, particularly those located in the executive subregion, plays a significant role in causing cognitive impairments observed in PD.

The current study has several limitations. First, the sample size utilized was comparatively small, thus future research endeavors involving more extensive cohorts are warranted to yield stronger and more reliable insights. Second, the MSV may be influenced by other factors such as calcium, copper, or lipid, although these effects are minor compared with those of iron. Third, the cross-sectional design of this study limits our ability to reach conclusions about the specific role of striatal iron in the progression of PD, therefore, more long-term studies are needed. Additionally, our current post-processing software requires manual segmentation to delineate the ROIs and lacks automatic segmentation functionality. In the future, we plan to select a more advanced post-processing software to achieve automatic segmentation and minimize result errors.

In summary, we documented the substantial accumulation of iron in the deep gray matter of patients with PD. The progressive pattern of iron deposition in the SN and GP might occur throughout the entire course of PD. Furthermore, there was a potential correlation between iron content in the SN, GP, and PT and certain clinical symptoms, such as motor impairments, anxiety, depression, and cognitive impairment. These findings offer a non-invasive biomarker for tracking disease progression and potentially contribute to a deeper understanding of the clinicopathological mechanisms underlying PD.

## Data Availability

The original contributions presented in the study are included in the article/Supplementary material, further inquiries can be directed to the corresponding author.
